# Overview of Serological Techniques for Influenza Vaccine Evaluation: Past, Present and Future

**DOI:** 10.3390/vaccines2040707

**Published:** 2014-10-13

**Authors:** Claudia Maria Trombetta, Daniele Perini, Stuart Mather, Nigel Temperton, Emanuele Montomoli

**Affiliations:** 1 Department of Molecular and Developmental Medicine, University of Siena, Via Aldo Moro, 53100 Siena, Italy; E-Mail: trombetta@unisi.it; 2 VisMederi srl, Enterprise in Life Sciences, Via Fiorentina 1, 53100 Siena, Italy; E-Mail: perini@vismederi.com; 3 Viral Pseudotype Unit, School of Pharmacy, University of Kent, Chatham Maritime, Kent ME4 4TB, UK; E-Mails: sm751@kent.ac.uk (S.M.); N.Temperton@kent.ac.uk (N.T.)

**Keywords:** correlates of protection, EMA criteria, antibody titres

## Abstract

Serological techniques commonly used to quantify influenza-specific antibodies include the Haemagglutination Inhibition (HI), Single Radial Haemolysis (SRH) and Virus Neutralization (VN) assays. HI and SRH are established and reproducible techniques, whereas VN is more demanding. Every new influenza vaccine needs to fulfil the strict criteria issued by the European Medicines Agency (EMA) in order to be licensed. These criteria currently apply exclusively to SRH and HI assays and refer to two different target groups—healthy adults and the elderly, but other vaccine recipient age groups have not been considered (*i.e.*, children). The purpose of this timely review is to highlight the current scenario on correlates of protection concerning influenza vaccines and underline the need to revise the criteria and assays currently in use. In addition to SRH and HI assays, the technical advantages provided by other techniques such as the VN assay, pseudotype-based neutralization assay, neuraminidase and cell-mediated immunity assays need to be considered and regulated via EMA criteria, considering the many significant advantages that they could offer for the development of effective vaccines.

## 1. Introduction

The human influenza virus is one of the most important infectious diseases in the world [1]. It is rooted in the past and has periodically decimated the world’s population since ancient times via pandemics.

Influenza virus infects all age groups but children and adults over the age of 65 are most at risk. Vaccination is recommended for these age groups, and also for anyone with high-risk conditions due to complications of influenza and those with chronic medical conditions (metabolic, cardiac, pulmonary or kidney diseases, as well as immunocompromised patients) [2,3]. Generally the same recommendation is extended for nursing home and health workers. 

Vaccines and antiviral drugs are the two primary methods of implementing influenza prophylaxis. They are the only effective ways to prevent infection and treat illness [4], and their availability plays a key role in the event of a pandemic. Evidence has been obtained from the relatively mild 2009 H1N1 influenza pandemic which demonstrated that vaccines and antiviral drugs were not readily available in time for more than 90% of the world’s population [5]. Antiviral drugs can give responsive protection against influenza virus and are useful for containment at the beginning of a pandemic, but in the long term, infection control depends on vaccination [6]. Vaccination remains the most efficacious method to control seasonal infections and the most important strategy to prepare for a possible pandemic [7].

The degree of protection elicited by vaccination depends on interplay between vaccine composition and circulating influenza viruses, the age of the vaccine recipient and their previous exposure to influenza. Currently, inactivated vaccines are the most effective means to counteract influenza infection [8]. They show a 60%–100% ability to prevent morbidity and mortality in low-risk target populations, such as healthy adolescents or adults, but may have little effect in younger (naïve) or older (decreased immune function) populations [9], as well as over time, due to low antigenic match [10].

In order for a vaccine to be marketed, it is necessary to evaluate its immunogenicity - the capacity of the vaccine to induce an immune response. Studies have shown that antibodies directed against viral haemagglutinin (HA) are an important correlate of protection [11,12].

Serological techniques commonly used to quantify influenza-specific antibodies include the Haemagglutination Inhibition (HI), Single Radial Haemolysis (SRH) and Virus Neutralization (VN) assays. HI and SRH are common, easy to perform and reproducible techniques, whereas VN is more laborious, less immediate, does not allow for simultaneous analysis of a large number of samples but is advantageous due to the possibility of detecting all functional antibodies that interfere with infection [13] and are of low antibody titres [14,15].

Recent studies have also been focused on the activity of neuraminidase (NA) [16,17] and on cellular immunity induced by vaccines [18,19]. 

Each vaccine needs to fulfil the criteria which include exclusively SRH and HI assays, issued by the Committee for Proprietary Medicinal Products (CPMP) in order to be licensed [20] (Table 1).

**Table 1:** Committee for Proprietary Medicinal Products (CPMP) criteria. Seroconversion (HI): if pre-vaccination serum is negative, then post-vaccination serum must have a titre ≥40; if pre-vaccination serum is positive, then at least a fourfold titre increase is required. Seroconversion (SRH): if pre-vaccination serum is negative, then post-vaccination serum haemolysis area must be ≥25 mm^2^; if pre-vaccination serum is positive, then there must be at least a 50% increase in haemolysis area. Seroprotection (HI): a serum sample is considered seroprotected when it shows an HI titre ≥40 or an SRH titre >25mm^2^. Seroconversion rate: proportion of subjects showing seroconversion. Seroprotection rate: proportion of subjects showing seroprotection.

18–60 Years	> 60 Years
Seroconversion rate > 40%	Seroconversion rate > 30%
Mean geometric increase > 2.5	Mean geometric increase > 2.0
Seroprotection rate > 70%	Seroprotection rate > 60%

The CPMP criteria refer to two different target groups—healthy adults between 18–60 and those over 60—but other age groups that may receive such vaccines have not been included. In 2006, the Committee on Immunization Practices (ACIP) updated their influenza vaccine recommendations and introduced categories such as children between 6–9 and 24–59 months, pregnant women, healthcare providers, patients with chronic disease and immunosuppressed patients [21]. Despite extensive research having been conducted in recent years to identify correlates of protection for these two age groups, little is currently known about protection in the event of hospitalization, secondary bacterial infections, chronic illness and even less about protective correlates in specific risk groups, even if they may be important for society [22,23].

## 2. Correlates of Protection

A correlate of protection is an important milestone in the development of a new vaccine but despite this, it still remains a very confused concept. Some definitions have been given, such as the one offered by Plotkin and Gilbert [24], which states that “a correlate reflects a statistical relation between an immune marker and protection but does not necessarily imply causal agency of the marker” and that proposed by Qin et al. [25], suggesting correlates predict “protection for new settings and describe the data requirements for rigorous validation of an immunological measurement at each level”. The concept is based on the immunogenic capacity of the vaccine to produce an antibody and/or cell-mediated immune response in its recipients [26].

Studies conducted by Hobson [11] and others [12,27,28] have established that HA antibody titre is correlated with protection against influenza infection. Generally an HI antibody titre of 40 is defined as 50% protective against influenza infection compared to HA titre < 10. 

Correlates of protection can be absolute or relative. Even if the ideal scenario is for a correlate to be absolute, meaning that protection is almost guaranteed by a definitive threshold of response (such is the case for diphtheria, tetanus and rubella), many correlates of protection are relative. In these instances, protection is usually conferred by a certain response level, but this can vary between vaccinated individuals and disease can occur in some vaccinees regardless of a theoretically protective correlate response [29]. For instance, with inactivated vaccines, the consensus derived from several studies is that an HI antibody level of 1:40 against HA should be considered protective [30]. However, this level is only 50%–70% protective so this correlate of protection should be regarded as relative rather than absolute [30]. 

Coudeville *et al.* [12] developed a model, using a meta-analytical approach, in order to estimate the level of protection against influenza associated with any HI titre level. The results suggest that the relationship between titre and protection is identified by a curve rather than a threshold. The clinical protection shows a progressive and significant increase at titres up to 100 (which included the common threshold of 1:40) but advantages become marginal beyond 150.

Pertaining to correlates of protection for influenza vaccines, it is also important to distinguish between young and elderly vaccinees, due to the fact that IgG serum antibodies only correlate well with protection for adults under the age of 50 [30]. Humoral immune responses raised against influenza viruses or related vaccines are mediated by several factors, such as age, the simultaneous presence of other diseases and the contemporaneous use of medicines that may affect immune function. Several studies have been conducted on the immune response to influenza vaccines and conflicting results were obtained. Some show that vaccination induces a lower HI antibody response in elderly compared to young recipients, while others report no discrepancy between age brackets or indeed report a contrary result. Protective immune indicators against influenza in at-risk groups must yet be defined since, even in this case, some studies have reported a reduced humoral response in risk groups, while other studies have shown that the humoral response is comparable to healthy control subjects [26].

It is also necessary to distinguish between vaccine efficacy and vaccine effectiveness. Often the distinction between these terms is ignored and they are used interchangeably, which can result in widespread confusion and misconception of “vaccine efficacy” [31]. In fact, “vaccine efficacy” is measured precisely as the ability of a vaccine to prevent disease in vaccinated individuals, with emphasis on the exact levels of vaccine-induced disease reduction [32]. “Vaccine effectiveness” refers to how well a vaccine protects against influenza when routinely used in the community, as opposed to in a randomized control trial. This is evaluated by observational studies and represents the reduction of infection frequency in vaccinated individuals compared to those who have not been vaccinated, assuming that the vaccine has induced said reduction [33].

Confusion also surrounds the topic of “surrogates of protection”. Consistent definitions have been published by both Plotkin and Quin, detailing a surrogate of protection: “as an immune marker that can substitute for the clinical end point and thus, can be used to reliably predict vaccine efficacy”. However, according to Quin, a surrogate may or may not be considered as a causal agent of protection, whereas Plotkin considers a surrogate of protection to be an immunological measurement performed when unable to ascertain a true correlate but stresses that there is no direct causality assumed with a surrogate [29]. Another relevant concept defines surrogates of protection as correlates able to predict the level of protective efficacy of a vaccine by comparing immunological measurements of vaccinated and unvaccinated individuals [34]. A general surrogate of protection needs to be adequately specific in several circumstances in order to be generalized to untested groups [35].

## 3. Haemagglutination Inhibition Assay

The HI assay is based on the ability of antibodies, if present in the serum, to prevent agglutination between erythrocytes and viral haemmaglutinin [36]. The antibody titre is expressed as the reciprocal of the highest serum dilution showing complete inhibition using 4 HAU units/25 µL or 8 HAU units/50 µL [8,37]. The starting dilution is generally 1:10 and the lower limit of a detectable antibody titre is 10. When the titre of antisera is under a detectable threshold, due to a low or non-existent amount of antibodies, this is conventionally expressed as 5, half the lowest detection threshold [38]. As previously mentioned, an antibody titre of 40 is generally considered as a protective threshold level, beyond which there is a 50% or greater reduction in the possibility of contracting influenza infection [11,39]. An HI titre equal to or greater than 40 is used as an immunological correlate of protection and is regarded as the best currently available parameter for predicting protection from natural infection, according to FDA guidelines for pandemic influenza vaccines [40]. The problem raised by Black *et al.* [41], however, is that this correlate is well defined in adults but not in children although it also applies to serology of samples from this age group. Children show a reduced capacity for cellular immunity and have little to no previous exposure to either influenza or vaccination. This situation necessitates the definition of a protective correlate specific to this category. More importantly, previous exposure to the influenza virus or vaccination can play a crucial role in reaching the protective threshold level in adults. In fact, even if all those who receive the vaccine are seroprotected (a subject is defined as seroprotected if the antibody level is above a certain cut-off [38]), this result may not correspond to a 50% vaccine-induced reduction of risk of influenza infection, due to the presence of protective antibodies in some subjects prior to vaccine administration. Therefore, an HI titre of 40 is not a threshold of protection induced by the vaccine but protection induced by antibodies in general [42]. This is the reason why, in vaccine assessment, seroprotection is complemented by the two further criteria, seroconversion and mean geometric increase which help discriminate high antibody titres prior to vaccination.

Black *et al.* [41] conducted a study in children aged between 6 and 72 months who had not previously been vaccinated in order to evaluate the relationship between HI antibody titre and clinical protection from influenza illness. The subjects were divided into 3 groups and each received two doses, one month apart, of either an MF59 Adjuvanted Trivalent Inactivated influenza Vaccine (ATIV, Fluad®, Novartis Vaccines, Siena, Italy), a subunit Trivalent Inactivated Influenza Vaccine (TIV, GlaxoSmithKline, Rixensart, Belgium) or a saline placebo. Clinical cases for influenza like illness were confirmed by reverse transcription polymerase chain reaction testing for influenza. For the recipients of the adjuvanted vaccine, results confirmed that a cut-off of 1:110 measured 21 days after the second vaccine dose could be used as a marker of 50% protection in the juvenile sample population. Titres of 1:215, 1:330 and 1:629 correspond to protection levels of 70%, 80% and 90% respectively (Table 2). Importantly, similar results could not be achieved in the TIV vaccine group. Therefore, it is evident that a higher HI titre is required in naïve subjects compared to those with a previous history of influenza infection in which other protective factors may come into play. A further consideration could be to assess whether the HI assay is indeed the best option to evaluate influenza vaccines for children [43].

Vaccination in children must also overcome other issues. In addition to defining protective correlates specific to the immune responses elicited by this age cohort, it is necessary to define the method and quality of vaccine administration, in addition to the most suitable vaccine evaluation techniques.

**Table 2:** Antibody cut-off level for clinical protection against influenza infection.

Antibody cutoff level for clinical protection	
Protection Level	Antibody Cutoff Level
50%	1:110
70%	1:215
80%	1:330
90%	1:629

## 4. Virus Neutralization Assay

The virus neutralization (VN) is a helpful assay for diagnostics and basic research that enables the observation of the humoral immune response against a virus [44]. 

In the VN assay, the highest serum dilution that induces a 50% inhibition of virus growth is identified as a neutralizing titre, based on the amount of virus in negative control wells [45]. The sample titre is defined as the maximum dilution factor at which antibodies can be identified. For instance, if there are detectable antibodies in a sample at a dilution of 1:40, the sample will have a titre ≥40 [46]. 

VN is a particularly useful technique for serology of avian strains of influenza A, and also influenza B viruses, as several studies have detailed the unsuitability of HI to detect antibody responses against these viruses [15,47,48,49,50]. Because of the insensitivity of detecting H5-induced antibody responses by HI, the quantification of functional neutralizing antibody responses is the analytical aim of VN immunogenicity studies [51]. 

In comparison to HI, the VN assay identifies a wide range of neutralizing antibodies because it detects antibodies that neutralize the virus via entry/replication inhibition in mammalian cells whereas HI only measures antibodies directed against viral haemagglutinin that act by preventing erythrocyte agglutination. As has been suggested in animal model studies, the prevention of infection is predicted by antibody-mediated neutralisation while disease prevention is correlated to HI [52,53,54,55,56]. 

Conventional neutralization tests are based on the inhibition of cytopathic effect in Madin-Darby Canine Kidney (MDCK) cell cultures, resulting in laborious and slow tests. A VN assay with microtitre plates, in combination with a downstream ELISA to detect virus-infected cells, is faster, producing results in just two days [37]. VN assays are also able to detect antibodies at low titres [14,52] and can distinguish between pre- and post-vaccination titres, especially in the instance of small (less than two-fold) differences between titres, when compared to HI [57]. 

One of the major drawbacks of the VN assay platform is the necessity to handle wild-type virus and the associated costs of high-level biocontainment facilities (*i.e.*, Biosafety Level 3 laboratory) when studying the serology of highly pathogenic strains, such as H5 and H7. In this instance, the use of influenza HA pseudotypes as surrogates for wild-type virus is a safer alternative, that may also have increased throughput capability and ease of standardization benefits (see Section 5). Other limitations of the VN assay include extensive training requirements for laboratory personnel, issues with throughput/simultaneous screening of large panels of sera and importantly, problems with standardising cell preparations, virus inoculations and incubation times [15,49]. Incidentally, inter-laboratory variation is significant with VN assays due to the lack of common reference protocols, discrepancies with assay endpoint determination and limited knowledge of correlates of protection. Currently, no protective correlates have been defined for VN in animal or human models, and due to the assay’s variability, a VN titre equivalent to an HI titre of 40 is highly specific for each antigen-laboratory combination and therefore cannot be generalized [57].

According to the World Health Organization (WHO), H5N1 infection can be confirmed by VN when one of the following criteria is met: a “fourfold or greater rise in antibody titre against A (H5N1) in paired sera (acute and convalescent) with the convalescent serum having a titre of 1:80 or higher or antibody titre of 1:80 or more in a single serum collected at day 14 or later after onset of symptoms and a positive result using a different serological assay” [58].

Scientific studies have uncovered a more complicated situation concerning VN, both in the case of H5N1 infection and in the case of other influenza infections, such as H1N1. Some studies of H5N1 employ a titre ≥80 as an efficacy endpoint for avian influenza vaccines [59,60], whereas others consider a seroprotection cut-off of 1:20 to be suitable, on the basis of correlation with an SRH area of 25 mm^2^ [13,45,51].

Based on previous research regarding H1N1, Allwin *et al.* [61] suggest a seropositive threshold of a 1:64 virus-neutralising serum dilution. Other studies agree in considering a titre ≥10 as the minimum detection limit and a titre ≥40 as a significant response [52,54,55]. The titre ≥10 could be interpreted as an effective indicator of population exposure to the virus by either natural infection or vaccination [46]. 

**Figure 1:**
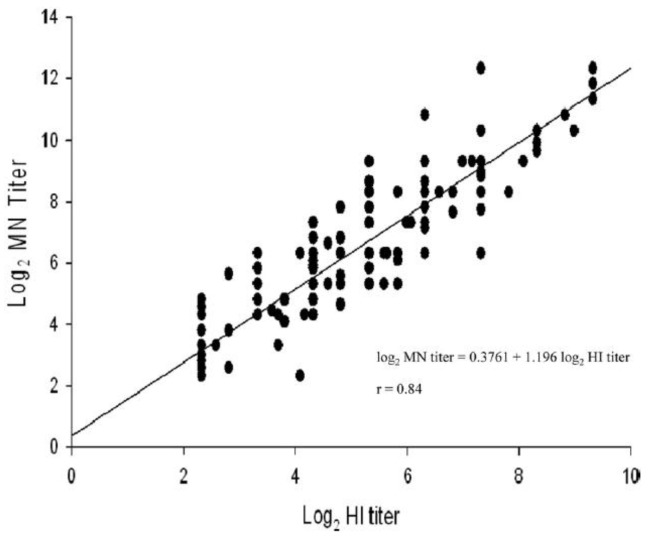
Correlation between antibody titres measured by VN and HI assays using sera from 87 confirmed cases of 2009 H1N1 virus. The results show a strong positive correlation between the two assay titres [52].

Regardless of the lack of clearly defined criteria for VN, many studies have shown that the assay correlates well with HI (Figure 1) [62,63,64] with the exception of H7 virus subtypes [64].

**Figure 2:**
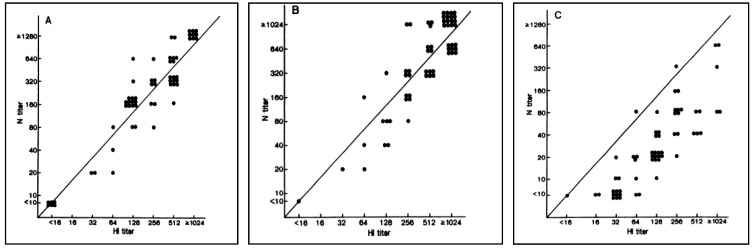
Relationship between HI and neutralizing antibody titres against A/Yamagata/12/86 (H1N1), A/Fukuoka/C29/85 (H3N2) and A/Shisen/2/87 (H3N2) [65].

Okuno *et al.* [65] have demonstrated a good correspondence between HI and VN titres in some circumstances and also report a heightened reliability of VN when compared to HI (Figure 2). They recorded both sets of titres raised against the seasonal influenza strains A/Yamagata/12/86 (H1N1), A/Fukuoka/C29/85 (H3N2) and A/Shisen/2/87 (H3N2). The results showed a good correlation between titres calculated for A/Yamagata/12/86 (H1N1) and A/Fukuoka/C29/85 (H3N2), but the VN titres were lower than those for HI against A/Shisen/2/87 (H3N2). An explanation of this is that, over recent years, most schoolchildren have received a multivalent vaccine including A/Yamagata/12/86 (H1N1) and A/Fukuoka/C29/85 (H3N2) strains, but not A/Shisen/2/87 (H3N2), since it was a candidate vaccine strain from 1988. 

The VN assay can be developed for any influenza A subtype [15] and has also been demonstrated for influenza B viruses. It is able to distinguish between viruses that belong to the same lineage, as well as to different sublineages [49].

Although VN assay results are not strictly necessary in obtaining Committee for Medical Products for Human Use (CHMP) approval, the EMA recommends the quantification of neutralizing antibodies able to inhibit viral attachment, entry and release of progeny virions [66]. This recommendation further reinforces the necessity to revise existing guidelines.

## 5. Pseudotype-Based Assays

**Figure 3:**
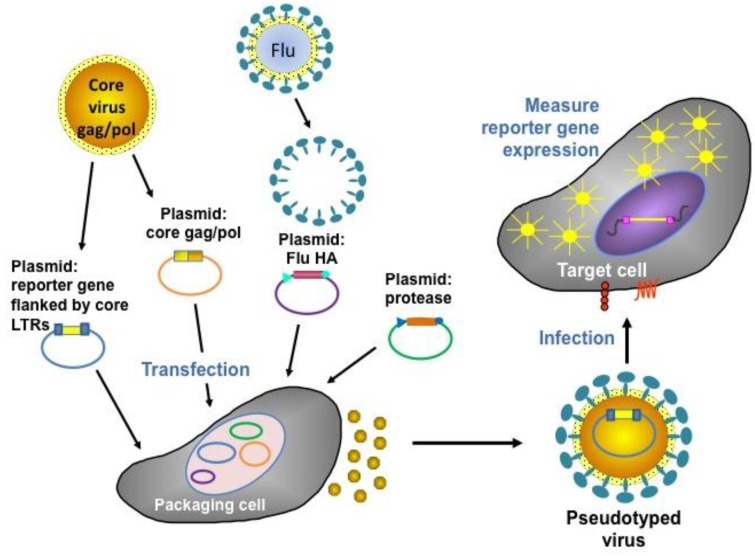
Representation of the production and assay of influenza HA pseudotyped retroviruses [72].

A pseudotype virus has the “core” of one virus (e.g., a retrovirus) and the outer “envelope” protein(s) of another (e.g., the HA/NA of influenza virus). The core virus has deletions in its genome making it replication-deficient (allowing it to be used under BSL1/2 containment), and harbours a reporter transgene (e.g., luciferase or Green Fluorescent Protein (GFP)). The envelope glycoprotein permits entry into susceptible target assay cells (e.g., Human Embryonic Kidney 293 T cells (HEK293T)/MDCK) via interaction with sialic acid. During target cell transduction, the pseudotype virus genome becomes integrated into the cell genome, resulting in reporter gene expression. Thus, the number of transduced cells can be accurately quantified (via a luminometer, fluorescent microscope or Fluorescence-Activated Cell Sorting (FACS)) and the subsequent inhibitory effects of functional antibodies (directed against the HA1 head and the HA2 stalk) in serum determined [67,68,69]. Figure 3 below shows a schematic representation of the production and assay of influenza HA pseudotyped retroviruses. Pseudotype production of Highly Pathogenic Avian Influenza (HPAI) strains (which have a polybasic cleavage site in the HA) routinely involves the transfection of three plasmids into 293T producer cells: retroviral *gag-pol* plasmid, HA-expressing plasmid, and retroviral vector plasmid incorporating the reporter gene. Additionally, for the production of Low Pathogenic Avian Influenza (LPAI) strains (single arginine at cleavage site), an additional plasmid expressing a protease (either Transmembrane Protease Serine 2 ((TMPRSS2) or Human Airway Trypsin-like Protease (HAT)) is necessary [70,71].

The pseudotype neutralization assay is also amenable to multiplexing provided that the two desired subtypes fall into different HA groups (HA group 1 comprises H1, H2, H5, H6, H8, H9, H11, H12, H13, H16 and H17; HA group 2 comprises H3, H4, H7, H10, H14 and H15). This has recently been successfully achieved with avian influenza strains H5 and H7 to study the immunogenicity of a bivalent H5/H7 chicken vaccine [73]. When evaluating sera for the presence and magnitude of neutralizing antibodies directed against two viruses (H1 and H3 for seasonal, H5 and H7 for pandemic, for example), there are distinct advantages to the use of a multiplex assay rather than individual subtype assays, which are in routine usage. In a multiplex format, inter-assay variability is likely to be reduced, since only a single serum dilution series needs to be performed and the same preparation of target cells (e.g. MDCK) is used for the two viruses. Also, the antibody response to one virus may serve as an internal “sero-standard” for the antibody response to the second virus and vice versa, as two separate luciferase reporters (firefly and renilla) are employed for this assay [73]. A pseudotype-based assay is more readily amenable to high-throughput processing of large serum panels, which will enable effective and informative larger-scale studies to be carried out. There are also beneficial safety and financial implications to the use of this assay, since antibody responses against two viruses are performed on the same serum samples with the entire process performed outside of high BSL facilities, which by their nature are expensive to maintain and require a high level of user training. The pseudotype-based neutralization assay is both “serum-sparing” and “antigen-sparing” as only 2–5 µL of serum is required per assay compared with the significantly larger volume required for traditionally employed influenza serology assays, and very small volumes of pseudotype virus (<1 µL virus/well) are required. It is therefore possible to measure antibody responses against large panels of sera more rapidly and more accurately than using traditional influenza serology assays (HI, VN, SRH). 

Pseudotype-based antibody assays have been shown to have broad utility for the detection of neutralizing antibody responses in avian and human sera, from natural infection and pre/post-vaccination against both avian and human influenza viruses [67,74,75,76,77,78,79,80]. Alberini *et al.* [74] undertook a comparative serological study using pseudotype neutralization, VN, HI and SRH. From this study, a pseudotype titre corresponding to a VN titre of 80 was extrapolated to give 1:357. Further large-scale comparative studies with both seasonal and pandemic vaccines are required to complement this.

Particular interest should be paid also to the potential of pseudotype neutralization assays to study antigenic evolution of influenza viruses. The continuous rapid evolution of influenza viruses, driven by error-prone replication and increasingly by immune pressure, significantly influences the sensitivity of available serological assays for this virus. It can also limit the efficacy of human and avian influenza vaccines and the susceptibility of these viruses to anti-viral drugs, via the emergence of drug resistance. As substitution rates are significantly higher in influenza HA and NA genes compared with internal genes, retroviral and lentiviral pseudotypes bearing HA and NA envelope glycoproteins devolved from the rest of the virus are ideal tools to monitor the effects of antigenic drift on serological outcomes, and can be used for accurate sequence-directed, highly sensitive, low-containment assays for measuring antibody responses against influenza HA. It is relatively straightforward to update the pseudotype-based HA neutralization assay to measure responses against newly emerging influenza viruses [69,71,74,78,81]. Upon availability of the viral RNA/cDNA, HA/NA genes can be sequenced, readily PCR-amplified and cloned, or custom synthesized, and retroviral pseudotypes prepared for use in neutralization assays. Site-directed mutagenesis of the HA can subsequently be used to “fine-tune” the neutralization assay in order to track the evolutionary progression of the circulating virus at the genetic, and more importantly for serology, at the antigenic level. Therefore, these assays can be continually and easily updated to measure the immunogenicity of current and new vaccines and therapeutics, and for sero-surveillance studies in new outbreak locations.

Another considerable advantage of pseudotype-based assays is the capacity for adequately evaluating the immune response induced by currently licensed influenza vaccines. 

In fact, antibodies elicited by such vaccines are predominantly haemagglutination-inhibition (HAI)-competent antibodies that target the globular head of HA, thus inhibiting pseudotype entry into target 293T cells [67,82]. These antibodies predominantly confer homosubtypic/strain-specific protection and only rarely confer heterosubtypic protection.

However, recent research by many groups is centered on the elicitation of antibodies directed against the stalk of the influenza HA that have been shown to confer broad protection across a range of subtypes. More specifically, pseudotype-based assays have been shown to be highly efficient for the measurement of broadly neutralizing antibodies directed against the HA2 stalk of influenza, making them ideal serological tools for the study of cross-reactive responses against multiple influenza subtypes with pandemic potential [81,83,84]. The microneutralization assay can also measure HA2 stalk responses, albeit with lower sensitivity. This is likely due to the fact that accessibility to the target epitope is limited by the tight packing of HA molecules in wild-type viruses as compared with pseudotype viruses [75]. The HI assay does not measure any HA2 antibodies. This underscores the importance of conducting comparative serological investigations to more accurately dissect the antibody response against novel vaccines and for sero-epidemiological studies, as each assay measures different (but occasionally overlapping) antibody responses [69,74,85]. 

## 6. Single Radial Haemolysis Assay

Single Radial Haemolysis (SRH) is a serological technique developed in 1975 [86] that combines the advantages of the Single Radial Diffusion (SRD) and HI assays [87]. This technique takes advantage of antibody diffusion within a gel for the determination of antibodies that might be present in analysed sera. The haemolysis, mediated by complement and induced by the antibody-antigen complex, produces easily identifiable “zones of haemolysis”, whose size is proportional to the concentration of anti-influenza antibodies present in the sera [88]. SRH has been used to detect antibodies not only against the influenza viral hemagglutinin but also against numerous other viruses such as coronaviruses, parainfluenza virus, Dengue virus and Japanese Encephalitis virus [89,90,91,92,93,94,95,96].

The greatest advantage of SRH is its safety as, unlike VN assays which require wild-type influenza virus, SRH is performed with inactivated virus. This aspect is particularly advantageous in the case of H5N1 because the serological tests can be safely carried out under BSL-2 containment [97]. SRH is inexpensive, rapid, reliable, reproducible and the quantitative, unbiased results are available after an overnight incubation [89,91,98,99,100,101]. Other significant advantages to the SRH assay include the ability to simultaneously and rapidly test a large number of samples without pre-treatment (excluding complement inactivation), and the requirement for only a small volume of sera [93]. 

For influenza A viruses, there is a good correlation between the results obtained by SRH and HI—an HI titre of 40 with human sera corresponds to an SRH titre of 19–33 mm^2^, while for influenza B viruses, SRH is consistently more sensitive than HI [102].

A study conducted by Morley *et al.* [88] shows that the results obtained with SRH and HI correlate strongly with those obtained with the VN, suggesting that the antibodies detected with these techniques may have the ability to neutralize the virus. In fact, given all these features, SRH seems to be a technique particularly suitable for detecting rises in influenza antibody titres [103].

**Figure 4:**
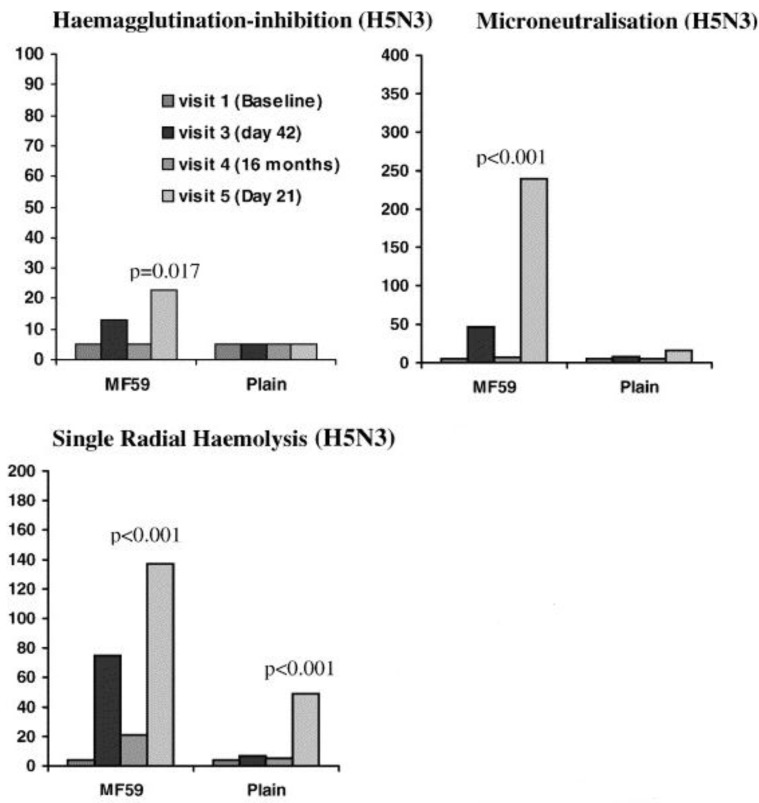
Antibody titres measured by HI, VN and SRH assays after a vaccination with a non-adjuvanted vaccine or an MF59-adjuvanted influenza A/Duck/Singapore/97 (H5N3) vaccine. At each visit, the HI assay was shown to underestimate antibody responses when compared to VN and SRH assays [106].

Following the emergence of avian influenza viruses capable of causing infection in humans, and limited use of HI assays for these viruses due to an underestimation of the human immune response raised against these pathogens [104,105,106] (Figure 4), SRH has been widely used as a sensitive and specific technique for the detection of human antibodies directed against avian influenza viruses in clinical trials [107,108,109]. 

This technique is officially recognized by the EMA and, in order for a vaccine to be licensed, it must meet two specific parameters for SRH in two age groups: adults from 18 to 60, and seniors over the age of 60. For the first age group, a vaccine can be licensed when showing a number of seroconversions or significant increase in HA antibody titre > 40%, or when the proportion of subjects with an area ≥25 mm^2^ is higher than 70%, or when the mean geometric increase > 2.5 [20] (Table 1). As for the elderly, the frequency of seroconversions or titre increase must be > 30%, or the proportion of subjects with a titre ≥25 mm^2^ higher than 60%, or a mean geometric increase of >2.0. 

Clinical studies conducted in children and/or adolescents show that, for these age groups, the parameters used to evaluate the immunogenicity of the vaccine are the same as for adults [110,111]. This situation again raises the need to revise the criteria for licensing vaccines for juvenile and adolescent age groups.

## 7. Neuraminidase Antibody Titration

Neuraminidase, in addition to HA, is the other major surface glycoprotein of the influenza viruses. The primary role of NA is apparent in the later stages of the virus life cycle and involves viral release and subsequent spread from infected cells [112]. NA is immunogenic but not all antibodies directed against NA are able to block its enzymatic function [113].

NA-targeted antibodies do not have neutralizing capacity defined as an ability to prevent infection, but are instead able to significantly reduce viral replication within, and egress from, host cells. These characteristics imply that NA affects both disease occurrence and severity, as well as the spread of infection [114,115]. Unfortunately, the role of antibodies against NA in immunity has not been studied sufficiently and conflicting results have been reported by various authors [116].

The factors that have limited the study of NA are increased surface expression of viral HA compared to NA (1:4/1:2) [117], antigenic competition leading to a greater HA response upon simultaneous antigen presentation [118] and the lack of a reliable detection technique [119].

The studies conducted on NA utilise a wide variety of techniques. Some studies have measured post-vaccination Neuraminidase Inhibition (NI) activity using a fluorescent substrate (MUN), and results reveal that data obtained using this type of test are rapid, sensitive, reproducible and quantitative, and allow for the distinction between infected and vaccinated animals [120].

Others have developed a high-throughput screening assay (Accelerated Viral Inhibition with NA AVINA) that is able to quantify viral replication through the evaluation of NA activity in cell culture wells. This technique has proven to be advantageous because it is not necessary to use specialized reagents and is relatively simple to perform. The required amount of virus for this assay is low and therefore it should be possible to carry out the assay with virus prepared in eggs, without necessitating a virus concentration step. Its characteristics make it particularly appropriate for predicting vaccine efficacy and may be extremely useful in establishing the immunogenicity of seasonal and pandemic influenza vaccines [121].

Currently, the most used technique for the quantification of antibodies against neuraminidase is the Enzyme-Linked Lectin Assay (ELLA). Originally developed by Lambré [122], the assay is based on the ability of neuraminidase to cleave sialic acid residues from a substrate (usually fetuin). The desialylation uncovers sugar residues present of the surface of fetuin, which are otherwise masked and unreachable—such residues are then recognized by peanut lectin labeled with HRP. However, the potential presence of antibodies against NA in experimental samples would inhibit this process.

Possible sources of NA for ELLA assay are: purified NA, mismatched viruses (containing HA not circulating in humans) and pseudotyped viruses.

The ELLA test is performed in microtitre plates, is able to evaluate specific antibody to NA and thus could be useful to investigate NA antigenic drift, as well as to measure the NA antibody response to vaccines. Safety, better sensitivity than the traditional thiobarbituric acid (TBA) assays and no hazardous reagents make it a promising assay for the study of NA antibody response [123,124]. 

However, the research conducted on NA is complicated by its instability and the lack of commercial vaccines with controlled NA activity [125]. Moreover, the instability of NA activity at certain concentrations, even a few months after production, and a variation of about 40-fold in the NA activity of different virus preparations made with similar strains have been observed, making the production of vaccines with a uniform content of NA difficult [126]. Additionally, the demand for a validated assay that can be used to measure antibody responses that can “neutralize” NA after vaccination is increasing [125].

A study conducted by Monto and Kendal [127] describes the effects linked to pre-existing antibodies directed against the NA during the onset of an influenza outbreak with a new HA subtype. In 1968, a Hong Kong type A influenza A (H3N2) virus arose, with a discrepant haemagglutinin but an unchanged neuraminidase compared to previously circulating Asian strains (H2N2). This study showed that individuals who had pre-epidemic antibodies against NA had a lower rate of influenza than those who lacked existing anti-NA antibodies. The relationship between the decrease of infection-rate and the increase of pre-existing anti-NA antibody levels provides evidence of how antibodies directed to NA could be protective during an influenza epidemic. 

The immunological response to NA could be of particular importance during an influenza pandemic where the majority of the population would be HA-naïve but could have previous immunity to NA [124]. In fact, the results of some studies have shown that prior infection with influenza H1N1 2009 (H1N1pdm09) could provide immune protection against H5N1 virus, as their NA protein head region sequences are more conserved [119,128]. In particular, antibodies directed against these conserved regions of NA could help to provide a significant level of protection against the disease [129]. A further study in naïve ferrets showed that cross-protection against H5N1 observed in adjuvanted seasonal trivalent or pandemic (H1N1) vaccines can be attributed to both HA and NA but the production of antibodies directed against viral NA provides a strong correlate of protection in this model [130].

The development of sufficiently powerful vaccines to maintain high levels of anti-NA antibodies in the population, to improve adaptive immune recognition during an antigenically similar influenza epidemic, is increasingly promising [127].

In 1974, Couch *et al.* [131] demonstrated that an inactivated, recombinant influenza virus vaccine was able to induce a monospecific antibody response to NA. This vaccine revealed itself to be antigenic, essentially non-reactogenic, and able to induce significant protection against disease. This protection was provided by a previous infection with an influenza virus that posessed an antigenically identical NA.

The currently administered whole-virus and subvirion vaccines are standardized purely on their HA content [132], despite containing both HA and NA glycoproteins, meaning that NA content variation is not controlled between vaccine batches [133].

It has been estimated that for the currently available vaccines, all TIVs contain NA antigen capable of inducing an antibody response directed against NA (antibodies that inhibit NA) while the Live Attenuated Influenza Vaccines (LAIV) induce a lower response against N1 and N2, compared to that obtained with the TIVs [16]. 

Although there are no analyses or content standards regarding NA antigen in approved influenza vaccines, studies show the need to include an immunogenic amount of NA in vaccines [134].

Adequate incorporation of NA in vaccines provides the potential to improve homologous immunization against influenza and to elicit expanded heterovariant immunity in the case of the emergence of an epidemic virus with unexpected antigenic changes [135], in other words the presence of NA could provide a stronger and broader immune response to influenza viruses. In addition, the incorporation of NA in vaccines could lead to a reduction of the HA dose necessary to induce a protective immune response [136].

Another advantage of NA is its slower antigenic evolution, which is evidenced by a greater stability in nature [137]. This characteristic implies that NA would be able to induce longer-lasting immunity than that provided by HA or conventional vaccines [138]. Whereas it is necessary to almost annually refresh the virus strains included in vaccines (since the antibody response is predominantly strain specific) [139], the advantage that the presence of an adequate amount of NA could offer should not be underestimated.

In addition, it has also been shown that when HA and NA are provided in equal amounts, and as purified proteins separated from other viral proteins, they are equivalent from an immunogenic viewpoint. One approach in order to elude “the antigenic competition” of the two antigens might be to even out the mixture, that is, to modify the amounts of both antigens in such a way that there is no competition. These changes may be of particular interest in the case of vaccines administered to humans, because antigen balancing may result in a more holistic immune response against influenza.

The use of purified, viral NA protein, would result in a lower toxicity than vaccines with whole, live or inactivated viruses and would not need adjuvants [138]. Kilbourne *et al.* [114] demonstrated the immunogenicity of a purified, non-adjuvanted influenza virus (N2) neuraminidase vaccine. A single dose of this preparation was non-reactogenic and immunogenic in primed human subjects.

In the light of recent studies and experience of the H1N1 pandemic in 2009, which epitomised the unpredictability of human influenza [139], a sustained research focus is required to investigate the protective effects and duration of antibodies elicited against NA [140].

## 8. Cell-Mediated Immunity

The immunity induced by current vaccines is predominantly based on antibodies capable of neutralizing pathogens [141]. The dominant role of antibodies and antibody data requests from the regulatory agencies in releasing an influenza vaccine have focused mainly on the antibody response, often overlooking cell mediated immunity [142] which is also necessary for defence against many pathogens [143].

Even if cell-mediated immunity does not appear to contribute significantly to the prevention phase of the infection, it plays an important role in viral clearance after influenza infection and may also prevent complications associated with influenza [10]. In particular, T lymphocytes play a crucial role in mediating the cellular immune response, by providing a helper antibody response and intervening directly in reducing viral replication [144].

In a mouse model, it has been shown that cytotoxic T lymphocytes (CTL) are protective against influenza viral infection and that cross-reactive CTL responses could be potentially protective against influenza viruses that present antigenic drift and that are not neutralized by antibodies [145].

In the context of T lymphocytes, CD8^+^ T cells play an important role in virus clearance [146] and are the principal mediators of what is termed “heterosubtypic immunity” understood as a type of protection against viruses that differ serologically, provided by the cellular response to cross-reactive epitopes [147].

Whilst knowledge of the role of CD4^+^ T cells in heterosubtypic immunity is still lacking [148], it has been shown that CD4^+^ T cells play a crucial role not only in clearance but also in the recall of CD8^+^ T cell responses, as well as in the maintenance of CD8^+^ memory cells [146].

The advantage of cell-mediated immunity (T cells) is the ability of this kind of immunity to target internal proteins common to heterologous viral strains thus providing the vaccine with the capacity to induce a protective immune response against a wider range of viral strains [149]. An integration of current vaccination strategies with a “T-cell based vaccine” strategy may turn out to be highly effective [150]. In particular, the NP protein represents the major target structure for cross-reactive CTL [151].

In addition to the features listed above, the results of a study conducted by Murasko *et al.* [152] also consider a further important aspect of cell-mediated response and the important role it could play in the control of influenza in an elderly population group. Assuming that neutralizing antibodies are the most significant defence prior to infection, cytokine T lymphocytes eradicate the virus-infected cells. In young people, both cell-mediated and immune responses provide a defence against influenza infection, while in the elderly, the antibody response is insufficient and the role of cell-mediated response becomes fundamental in the eradication of the virus.

Beyond neutralizing antibodies, non-neutralizing antibodies have been the object of recent studies, due to their ability to provide some protection against influenza infection [153,154]. Jegaskenda *et al.* [155] developed a novel assay able to assess the specificity and function of antibody dependent cellular cytotoxicity (ADCC) specific to influenza. The ADCC antibodies against influenza virus have been identified also in absence of detectable neutralizing antibodies.

Therefore, the vaccines that promote cellular immunity may be an important alternative in combating potentially lethal and highly pathogenic influenza viruses, as well as providing help in preventing an influenza pandemic by promoting strong memory CTL activity, in conjunction with the antibody-based approach already underway [149].

## 9. Conclusions

The influenza virus has decimated populations throughout the world since ancient times [156] and still remains one of the most serious and persistent health problems for humanity, due to high rates of morbidity and significant economic cost [157]. 

The characteristic that makes influenza viruses exceptional is their ability to evade host immunity and cause recurrent annual outbreaks, and at irregular intervals major global pandemics, due to the introduction of antigenically new viruses in a immunologically naïve human population [158].

Vaccination has historically been a pillar for infection control [159] and provides both direct and indirect effects [160]. The direct effects are exhibited in a decreased susceptibility towards the disease by recipients, which implies a reduction in the probability of disease contraction. Indirect effects include a reduced risk of disease in both vaccinated and unvaccinated persons. Therefore, when an uninfected person comes into contact with someone infected, the likelihood of disease transmission is lower, compared to in the absence of vaccination.

In order for a vaccine to be licenced, it must meet at least one of three criteria required by the EMA, depending on the age group [20]. Children and adolescents represent a crucial point because the vaccination is also recommended for these cohorts [21], but they are not yet included as age groups in the official criteria used to authorize a vaccine. 

The question raised by Black *et al.* [41] is an essential starting point in analysing the current situation regarding juvenile vaccination, as data for this age cohort showed that the conventional HI titre of 40 provided only 22% protection from clinical infection. It is increasingly necessary to have adequate criteria specific for children, in order to licence a vaccine. Also, given that children and adolescents are widely vaccinated in the event of a pandemic, it would be optimal to administer a vaccine type and dosage specific to this age group, so as to curtail outbreak severity.

The criteria currently in use require revision in light of the aforementioned situation, and because criteria validity may be discrepant between seasonal and pandemic influenza vaccines [161]. Furthermore, it has yet to be confirmed whether correlates of protection provided by egg-derived vaccines can also be applied to cell-culture derived vaccines [162].

Another aspect that should not be underestimated concerns vaccine formulations and in turn, the related serological correlates. At present, correlates of protection for influenza vaccines lack adequacy with regards to LAIV vaccines, and thus guidelines cannot be applied for these formulations [123,163].

Given the importance of vaccination in order to protect humanity from disease, a critique of the current situation is necessary and criteria required for official vaccine dissemination need to be updated. Malleability of criteria between vaccine types and target populations would also be desirable, given the complicated state of play within vaccine administration. Each individual target population has different immunologic conditions and varying levels of prior exposure to the virus, making it inevitably necessary to expand upon the two age groups currently considered in official EMA criteria. Besides extending vaccination to additional age groups, the technical support provided by the VN, NA activity and Cell-Mediated Immunity (CMI) could prove useful. VN assays using live virus and pseudotypes would both need to be regulated so that these assays can be officially used for vaccine licencing, in order to serologically prepare for the emergence of new influenza viruses. The NA activity and CMI require further study, considering the significant benefits they could offer to vaccine effectiveness. 
